# Verification-phase tests show low reliability and add little value in determining V˙O_2_max in young trained adults

**DOI:** 10.1371/journal.pone.0245306

**Published:** 2021-01-11

**Authors:** Jonathan Wagner, Max Niemeyer, Denis Infanger, Timo Hinrichs, Clement Guerra, Christopher Klenk, Karsten Königstein, Christian Cajochen, Arno Schmidt-Trucksäss, Raphael Knaier

**Affiliations:** 1 Department of Sport, Exercise and Health, University of Basel, Basel, Switzerland; 2 Department Medicine, Training and Health, Institute of Sport Science and Motologie, Philipps-University Marburg, Marburg, Germany; 3 Centre for Chronobiology, Psychiatric Hospital of the University of Basel, Basel, Switzerland; 4 Transfaculty Research Platform Molecular and Cognitive Neurosciences, University of Basel, Basel, Switzerland; University of Bourgogne France Comté, FRANCE

## Abstract

**Objective:**

This study compared the robustness of a $\dot{V}O_{2}$-plateau definition and a verification-phase protocol to day-to-day and diurnal variations in determining the true $\dot{V}O_{2}\max$. Further, the additional value of a verification-phase was investigated.

**Methods:**

Eighteen adults performed six cardiorespiratory fitness tests at six different times of the day (diurnal variation) as well as a seventh test at the same time the sixth test took place (day-to-day variation). A verification-phase was performed immediately after each test, with a stepwise increase in intensity to 50%, 70%, and 105% of the peak power output.

**Results:**

Participants mean $\dot{V}O_{2}peak$ was 56 ± 8 mL/kg/min. Gwet’s AC_1_ values (95% confidence intervals) for the day-to-day and diurnal variations were 0.64 (0.22, 1.00) and 0.71 (0.42, 0.99) for $\dot{V}O_{2}$-plateau and for the verification-phase 0.69 (0.31, 1.00) and 0.07 (−0.38, 0.52), respectively. In 66% of the tests, performing the verification-phase added no value, while, in 32% and 2%, it added uncertain value and certain value, respectively, in the determination of $\dot{V}O_{2}\max$.

**Conclusion:**

Compared to $\dot{V}O_{2}$-plateau the verification-phase shows lower reliability, increases costs and only adds certain value in 2% of cases.

## Introduction

The maximum volume of oxygen uptake per minute ($\dot{V}O_{2}\max$) is the gross criterion for endurance performance and is determined by cardiopulmonary exercise testing (CPET) [1]. The achievement of $\dot{V}O_{2}\max$ is usually accepted when a distinct plateau of the $\dot{V}O_{2}$ work rate relationship in the severe intensity domain ($\dot{V}O_{2}$-plateau) occurs [2, 3]. However, the criteria at which a $\dot{V}O_{2}$-plateau is present are frequently misunderstood [4]. Furthermore, often less than the half of participants showed a plateau at the end of a continuous incremental exercise test [4–6]. As a consequence of the low $\dot{V}O_{2}$-plateau incidences, secondary exhaustion criteria such as a maximum blood lactate concentration, maximum respiratory exchange ratio or maximum heart rate have been developed and frequently used for the diagnoses of $\dot{V}O_{2}\max$ [2, 7, 8]. They can be used to reduce the magnitude of a potential underestimation of $\dot{V}O_{2}\max$ [5, 9]. However, the values of these criteria vary considerably between participants [10] and are affected by the exercise protocol used [11]. Consequently, even if rather high and age-adjusted secondary exhaustion criteria are used an underestimation of $\dot{V}O_{2}\max$ cannot be excluded [12].

To overcome this problem, a method called “verification-phase” is currently being promoted as a tool to detect true $\dot{V}O_{2}\max$ [3, 8, 13]. The basic idea of this concept is to provoke a $\dot{V}O_{2}$-plateau by inducing a constant exercise bout at a power output which is higher than the peak power output of a previously performed regular CPET (i.e. verification phase) [3, 8]. Compared to the $\dot{V}O_{2}$-plateau incidence during incremental exercise higher incidences of supposedly successful verified $\dot{V}O_{2}\max$ were reported [14, 15] suggesting an advantage of the verification phase compared to the classical plateau criterion. However, especially in CPETs with high incremental rates leading to exhaustion in about 8–12 minutes supra-peak work rates cannot be sustained for sufficient durations to allow $\dot{V}O_{2}$ to rise to the maximum value, as recently described [16]. High incremental rates are especially applied to endurance-trained participants to ensure that the presumed optimal duration for $\dot{V}O_{2}\max$ testing will not be exceeded [6, 17, 18]. This may limit the validity of supra-peak verification exercise to provoke a $\dot{V}O_{2}$-plateau in this cohort, which raises to the question whether supra-peak verification exercise can add additional value in determining $\dot{V}O_{2}\max$ compared to the $\dot{V}O_{2}$-plateau occurrence of the incremental phase.

Furthermore, methods and criteria are necessary for reliable measurement of endurance performance in athletes [19]. However, the robustness of the $\dot{V}O_{2}$-plateau and the ability of the verification bout to confirm or disprove the achievement of $\dot{V}O_{2}\max$ against day-to-day and diurnal variations in endurance performance have been rarely tested. Three studies checked the test-retest reliability of verification $\dot{V}O_{2}$ values [15, 20, 21]. Despite high correlations between the test re-test values found in the latter studies this does not prove that the verification or falsification of $\dot{V}O_{2}\max$ is reliable. The latter requires that $\dot{V}O_{2}peak$ values of incremental tests are consistently confirmed or disproved by the verification phase, which has never been checked. Furthermore, CPET's are often performed at different times of day. Therefore, criteria for the diagnosis of $\dot{V}O_{2}\max$ must be robust against diurnal variations [22]. However, the diurnal variability of the $\dot{V}O_{2}$-plateau and the verification procedure is unclear.

The aims of this study were to investigate the reliability of a $\dot{V}O_{2}$-plateau and a verification-phase protocol in male and female athletes regarding diurnal and day-to-day variations. A further aim was to analyse the percentage of tests in which performing a verification-phase added certainty in the determination of $\dot{V}O_{2}\max$.

## Material and methods

### Study design

This study was conducted between December 2016 and May 2018 in the laboratory of the Department of Sport, Exercise and Health of the University of Basel, Switzerland. The study was carried out in accordance with the recommendations of the “Ethikkommission Nordwest- und Zentralschweiz” and was approved by the same ethics committee (EKNZ 2016–01572). All participants gave written informed consent in accordance with the Declaration of Helsinki. Participants performed CPET at 7:00 am, 10:00 am, 1:00 pm, 4:00 pm, 7:00 pm, and 9:00 pm (i.e., diurnal variation). The sequence of the test times was equal for all participants but the time of the first exercise test was randomized. The seventh exercise test was always performed at the same time of day as the sixth test (i.e., day-to-day variation). Therefore, the majority of exercise tests were separated by 27 hours and the minimum was 26 hours.

### Participants

The participants were recruited by postings and posters. Inclusion criteria were age between 18–40 years. Exclusion criteria were health-related problems that are contraindicated for exercise testing as well as the use of medication that affect endurance performance. Because performing multiple exercise tests may lead to training effects in untrained individuals [23] only trained participants performing regular endurance training were included in the study. In detail, participants with a $\dot{V}O_{2}peak$ < 50 ml/(kg/min) for males and < 44 ml/(kg/min) for females were in the first incremental test were excluded from the study. This criterion is based on the 95th percentile of The American College of Sports Medicine reference values for $\dot{V}O_{2}\max$ (i.e., 56 ml/kg/min for males and 50 ml/kg/min for females). However, it may be possible that a participant is randomized to perform his/her first CPET at a time of the day at which his/her performance is at the nadir and therefore slightly under the inclusion threshold. He/she would therefore be excluded, although he/she might have reached the inclusion criteria if randomized to another time of the day for the first test session. Therefore, we reduced the criterion for $\dot{V}O_{2}\max$ by 10% which is based on the expected maximum diurnal variation of $\dot{V}O_{2}\max$ during the day [22]. On the first test day, participants were physically examined by a physician; 12 channel resting electrocardiography was performed and medical history was assessed. Before each CPET, body mass (kg) and body fat mass (kg) were measured with four-segment bioelectrical impedance analyses (Inbody 720, Biospace, Seoul, South Korea). The participants were instructed to refrain from caffeine, alcohol, and sports for the duration of the study.

### Initial exercise test phase

The exercise test was performed on a bicycle ergometer (Sport Excalibur, Lode Medical Technology, Groningen, The Netherlands) under standardized laboratory conditions (air humidity 40–55%, room temperature 20–22°C). On the first testing day, saddle and handlebar positions were fitted according to individual preferences. The values were noted and exactly replicated on subsequent testing days. For male/female participants the exercise protocol consisted of 75/50 W for five minutes (warm-up), a linear increase of workload with 25/20 W/min until exhaustion, and 75/50 W for ten minutes (regeneration). During all tests strong verbal encouragement was given. The highest mean of consecutive $\dot{V}O_{2}$ measures during 30 seconds was determined as $\dot{V}O_{2}peak$. Gas exchange was measured breath-by-breath (MetaMax 3B, Cortex Biophysik GmbH, Leipzig, Germany). Furthermore, heart rate was measured with 12 channel electrocardiography (Custo med GmbH, Ottobrunn, Germany) and also with a heart rate belt (Polar T-34, Polar Electro Europe AG, Zug, Switzerland). Rating of perceived exertion was assessed according to the 6–20 Borg scale. Blood lactate concentration was measured at rest, immediately after exhaustion, and at minutes one, three, five and ten of the regeneration phase. Blood samples were analysed immediately after the exercise test (SuperGL Ambulance, Hitado Diagnostic Systems, Moehnesee, Germany).

### $\dot{V}O_{2}$-plateau

A $\dot{V}O_{2}$-plateau was defined as Delta-$\dot{V}O_{2}$ < 125 mL between the oxygen uptake in the last 25 W and the second-to-last 25 W of the CPET [4]. Based on the assumption that $\dot{V}O_{2}$ increases approximately 10 mL/min per Watt in the submaximal intensity domain [24] a $\dot{V}O_{2}$ increase of 250 mL/min is expected between the last 25 W and the second-to-last 25 W. The cut-off was chosen at 50% of the expected increase of $\dot{V}O_{2}$ as recommended by Niemeyer et al. [4].

### Exercise protocol verification-phase

After the regeneration phase, workload was set to 50% of peak power output (PPO) achieved during CPET for two minutes, then increased to 70% of PPO for one minute, followed by an increase to 105% of PPO until exhaustion. Afterwards, participants performed a cool-down phase for three minutes. $\dot{V}O_{2}\max$ verification was accepted if the verification-$\dot{V}O_{2}$ was ±3% of the $\dot{V}O_{2}\max$ from the initial phase of CPET [25].

### Data analysis

For our analyses, we used SPSS Statistics (Version 24, IBM, Armonk, NY, USA) and for graphics R (Version 3.3.1, R Foundation for Statistical Computing, Vienna, Austria), respectively. No prior sample size calculation was performed, due to insufficient preliminary data. Descriptive statistics were used to present the diurnal and day-to-day variation in the $\dot{V}O_{2}$-plateau and verification-phase. In detail, scatterplots were used to show the “Delta-$\dot{V}O_{2}$” (i.e., difference in $\dot{V}O_{2}$ during the last 25 W of the CPET to the second-to-last 25 W) and the percentage of verification-$\dot{V}O_{2}$. The latter was calculated by dividing the $\dot{V}O_{2}peak$ of the verification-phase by the $\dot{V}O_{2}peak$ from the incremental test and expressed as percentage). We used Gwet’s agreement coefficient (Gwet’s AC_1_) [26] to quantify agreement between day-to-day variation and diurnal variation, because it is well known that common reliability measures such as Cohen’s kappa can exhibit low values in the case of severe imbalance of categories, even if absolute agreement is high [27]. Gwet’s AC_1_ is more robust against trait imbalance and shows plausible values [26]. A value of 0 signifies no agreement and a value of 1 signifies perfect agreement. For the day-to-day variation, we assessed if there was an agreement between the appearance (“1”) or non-appearance (“0”) of a $\dot{V}O_{2}$-plateau between the two tests performed at the same time of the day (i.e., sixth and seventh test). The same procedure was performed for the verification-phase. Due to technical problems, $\dot{V}O_{2}$ during the verification-phase was only available in 17 out of 18 participants (i.e. 34 tests). Furthermore, blood samples at termination of the incremental phase could not be collected in 5 out of the 126 tests.

Maximum physical performance, and therefore $\dot{V}O_{2}peak$, varies over the course of a day [22]. However, it is unclear whether this is caused by physiological mechanisms or variations in motivation and pain tolerance [22]. If the diurnal variation in $\dot{V}O_{2}peak$ is caused by a varying upper limit of O_2_ transportation and utilisation, a plateau should consistently occur or not occur to be a reliable criterion for the diagnosis of $\dot{V}O_{2}\max$. In contrast, if the diurnal variation in $\dot{V}O_{2}peak$ is caused by variations in motivation and pain tolerance a plateau should occur at the highest $\dot{V}O_{2}peak$ tests only. Since the reasons for the diurnal variation of $\dot{V}O_{2}peak$ are unclear, we only compared tests with similar $\dot{V}O_{2}peak$ to check for the diurnal variability of the $\dot{V}O_{2}$-plateau and the verification procedure. In detail, we calculated the typical measurement error [19] and excluded tests from the analyses in which $\dot{V}O_{2}peak$ differed more than twice the typical measurement error (i.e., 200 mL/min) from the $\dot{V}O_{2}peak$ achieved in the tests with the highest $\dot{V}O_{2}peak$. The same procedure was performed for the verification-phase. The chance of verifying $\dot{V}O_{2}\max$ during the verification-phase is expected to depend on the exercise duration at supra-peak power output [16]. Therefore, we additionally calculated the Pearson’s correlation between the duration at supra-peak power output (i.e. 105% PPO) and the difference between $\dot{V}O_{2}$ from the initial phase of CPET and verification-$\dot{V}O_{2}$.

**Fig 1:**
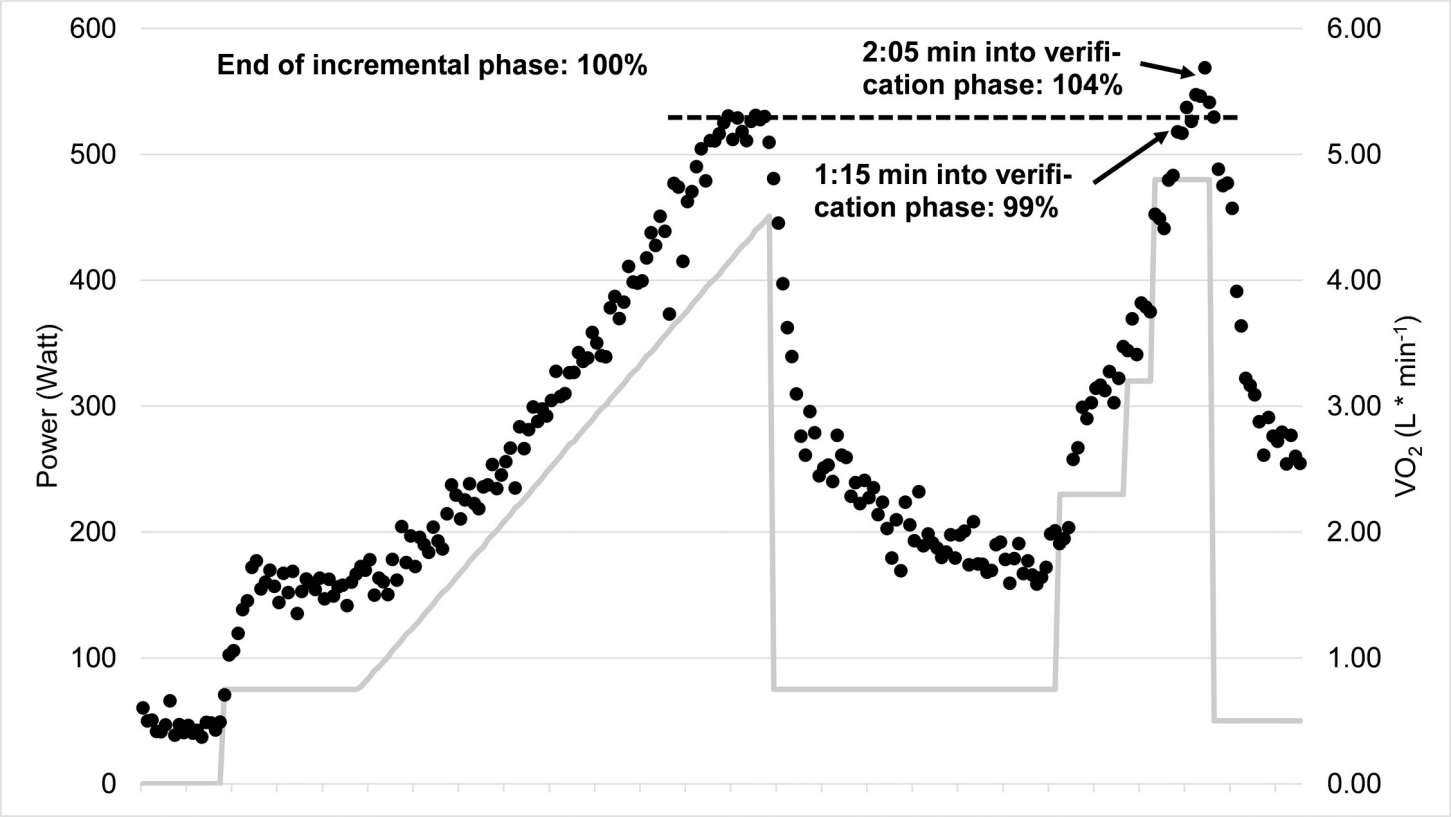
$\dot{V}O_{2}$ and work rate profile during a supra-peak power verification-bout in an individual, which was able to sustain the verification bout long enough to disprove the achievement of $\dot{V}O_{2}\max$ in the incremental test.

To investigate the additional value by performing a verification-phase on $\dot{V}O_{2}\max$ determination we calculated the percentage of tests for each of the following three conditions: (1) no added value, (2) uncertain added value, and (3) certain added value. No added value was defined as $\dot{V}O_{2}$-plateau present, irrespective of verification-$\dot{V}O_{2}$, which by itself indicates that $\dot{V}O_{2}\max$ was reached, or as no $\dot{V}O_{2}$-plateau was present and verification-$\dot{V}O_{2}$ < 97% of $\dot{V}O_{2}peak$ from initial phase of CPET. Uncertain added value was defined as no $\dot{V}O_{2}$-plateau present and verification-$\dot{V}O_{2}$ of 97–103% of $\dot{V}O_{2}peak$ from initial phase of CPET. This case was defined as uncertain because there are two options: first $\dot{V}O_{2}\max$ was reached during the initial phase of CPET and confirmed by the verification-phase; second $\dot{V}O_{2}\max$ was not reached during the initial phase of CPET, but the duration of the verification-phase was too short to disprove low $\dot{V}O_{2}\max$ (see Fig 1). Certain added value was defined as no $\dot{V}O_{2}$-plateau present and verification-$\dot{V}O_{2}$ > 103% indicating that the verification-phase was able to disprove low $\dot{V}O_{2}\max$.

## Results

### Participant characteristics

Twenty-seven participants were assessed for eligibility. Six participants did not meet the inclusion criteria regarding the $\dot{V}O_{2}\max$, one was excluded for medical reasons, and two participants had to be excluded due to technical measurement problems. Finally, eleven males and seven females were included in the study. Mean participant age, height, body mass, and body mass index were 28 ± 5 years, 174.6±7.6 cm, 69.7 ± 8.3 kg, and 22.8 ± 1.5, respectively.

### Descriptive statistics

Descriptive findings from the seven incremental tests and the corresponding verification phases are shown in Table 1.

**Table 1:** Findings from the incremental tests and corresponding verification phases including the absolute and percentage rate of $\dot{V}O_{2}peak$ confirmation by the $\dot{V}O_{2}$-plateau, secondary exhaustion criteria and the verification procedure.

		7:00	10:00	13:00	16:00	19:00	21:00	Re-Test
**Incremental Phase**	**Peak Power (W)**	341.7 ± 64.4	347.1 ± 63.5	346.3 ± 66.8	346.2 ± 62.3	350.9 ± 65.6	341.5 ± 63.4	347.6 ± 64.9
	**TTE (min)**	12.1 ± 1.8	12.3 ± 1.8	12.3 ± 1.9	12.3 ± 1.7	12.5 ± 2.0	12.2 ± 1.9	12.3 ± 1.8
	$\dot{V}O_{2}peak$ **(L/min)**	3.95 ± 0.81	3.96 ± 0.82	3.90 ± 0.73	3.92 ± 0.79	4.04 ± 0.77	3.95 ± 0.78	3.96 ± 0.83
	$\Delta \dot{V}O_{2}$ **(mL/min)**	169.9 ± 71.8	159.4 ± 96.6	179.1 ± 106.4	165.7 ± 78.4	177.8 ± 74.7	151.3 ± 68.5	184.8 ± 60.0
	**RERpeak**	1.23 ± 0.10	1.20 ± 0.09	1.22 ± 0.10	1.21 ± 0.09	1.28 ± 0.28	1.23 ± 0.09	1.22 ± 0.09
	**HRpeak (bpm)**	185.0 ± 7.4	187.9 ± 9.8	187.5 ± 8.3	186.8 ± 9.3	187.4 ± 7.9	187.5 ± 8.7	185.9 ± 7.5
	**BLCpeak (mmol/l)**	10.1 ± 2.5	11.9 ± 2.4	12.1 ± 2.6	11.3 ± 2.4	12.6 ± 2.5	11.4 ± 2.9	11.6 ± 2.3
	$\Delta \dot{V}O_{2}$ **< 125 (n (%))**	5/18 (27.8%)	6/18 (33.3%)	5/17 (29.4%)	6/17 (35.3%)	2/17 (11.8%)	7/18 (38.9%)	3/18 (16.7%)
	**RERpeak > 1.1 (n (%))**	16/18 (88.9%)	16/18 (88.9%)	16/18 (88.9%)	17/18 (94.4%)	16/18 (88.9%)	17/18 (94.4%)	17/18 (94.4%)
	**HRpeak > 95% 210-age (n (%))**	17/18 (94.4%)	17/18 (94.4%)	16/18 (88.9%)	17/18 (94.4%)	17/18 (94.4%)	17/18 (94.4%)	17/18 (94.4%)
	**BLCpeak > 10 mmol/L (n (%))**	8/15 (53.3%)	15/18 (83.3%)	13/18 (72.2%)	13/18 (72.2%)	14/17 (82.4%)	11/17 (64.7%)	13/18 (72.2%)
**Verification Phase**	**TTE (min)**	1.13 ± 0.29	1.13 ± 0.33	1.13 ± 0.33	1.25 ± 0.35	1.21 ± 0.29	1.17 ± 0.40	1.18 ± 0.43
	$\dot{V}O_{2}peak$ **(L/min)**	3.74 ± 0.78	3.69 ± 0.74	3.67 ± 0.68	3.82 ± 0.82	3.88 ± 0.76	3.79 ± 0.76	3.79 ± 0.84
	$\dot{V}O_{2}peak$ **VER/INC (%)**	94.8 ± 5.1	94.7 ± 4.4	95.7 ± 4.2	97.4 ± 4.5	95.9 ± 4.4	96.1 ± 3.8	94.2 ± 5.2
	$\dot{V}O_{2}peak$ **VER/INC < 97% (n (%))**	11/16 (68.8%)	12/17 (70.6%)	10/17 (58.8%)	7/18 (38.9%)	10/18 (55.6%)	9/18 (50%)	12/17 (70.6%)
	$\dot{V}O_{2}peak$ **VER/INC 97–103% (n (%))**	5/16 (31.2%)	5/17 (29.4%)	7/17 (41.2%)	9/18 (50%)	7/18 (38.9%)	9/18 (50%)	5/17 (29.4%)
	$\dot{V}O_{2}peak$ **VER/INC >103% (n (%))**	0/16 (0%)	0/17 (0%)	0/17 (0%)	2/18 (11.2%)	1/18 (5.6%)	0/18 (0%)	0/17 (0%)
	**No value (n (%))**	11/16 (68.8%)	14/18 (77.8%)	13/17 (66.5%)	12/17 (70.6%)	12/18 (66.7%)	11/18 (61.1%)	12/17 (70.6%)
	**Uncertain value (n (%))**	5/16 (31.2%)	4/18 (22.2%)	4/17 (23.5%)	4/17 (23.5%)	5/18 (27.8%)	7/18 (38.9%)	5/17 (29.4%)
	**Certain value (n (%))**	0/16 (0%)	0/18 (0%)	0/17 (0%)	1/17 (5.9%)	1/18 (5.6%)	0/18 (0%)	0/17 (0%)

TTE, time to exhaustion; $\dot{V}O_{2}peak$, highest oxygen uptake; $\Delta \dot{V}O_{2}$, difference between the final and second-to-final 25 W, RERpeak, highest respiratory exchange ratio; HRpeak, highest heart rate; BLCpeak, highest blood lactate concentration. Note that a $\Delta \dot{V}O_{2}$ < 125 indicates the occurrence of a $\dot{V}O_{2}$-plateau.

**Fig 2:**
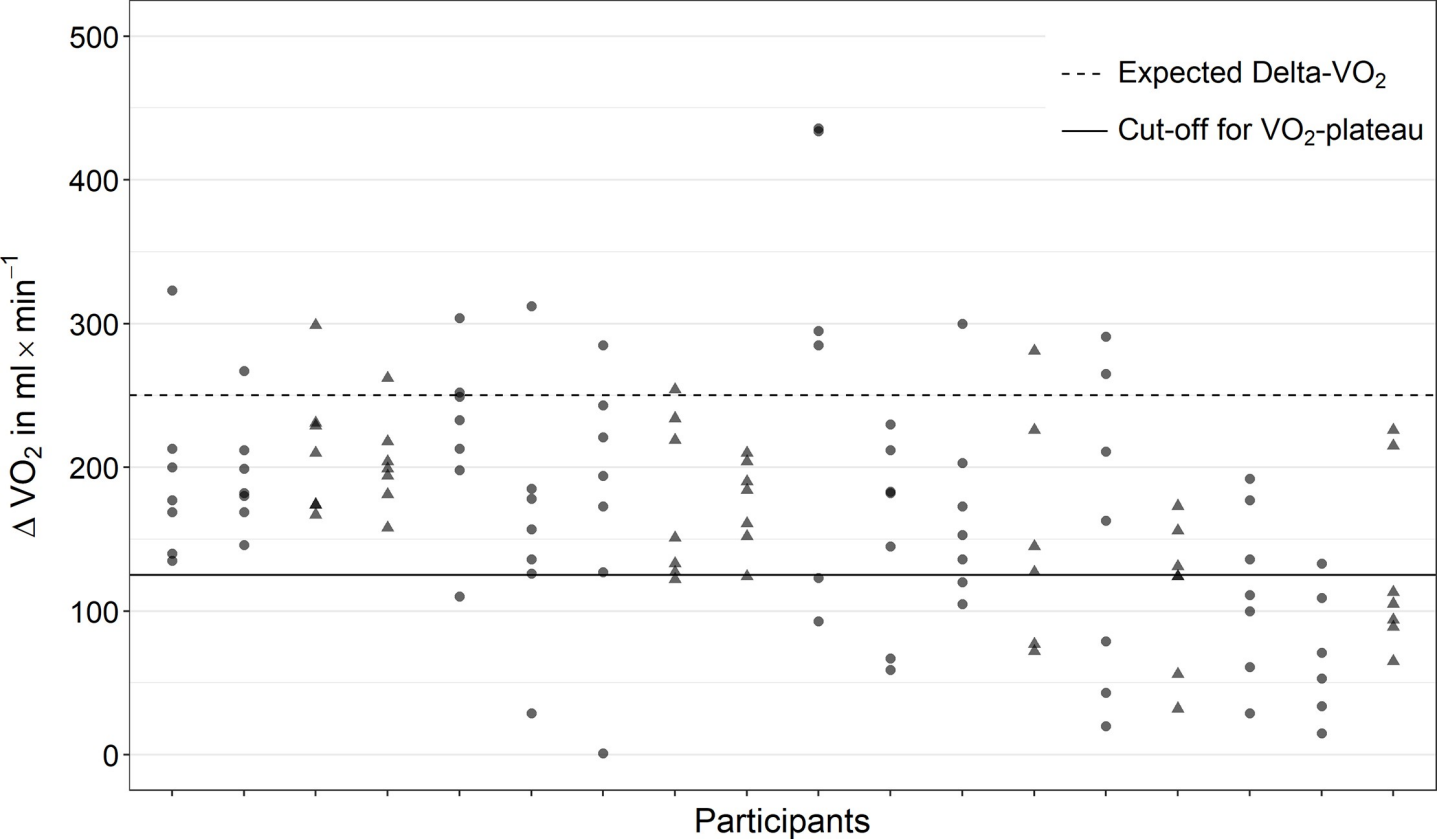
Difference in $\dot{V}O_{2}$ ($\Delta \dot{V}O_{2}$) during the last 25 W of the CPET to the second-to-last 25 W in all tests performed by the participants. Circles = males; triangles = females.

**Fig 3:**
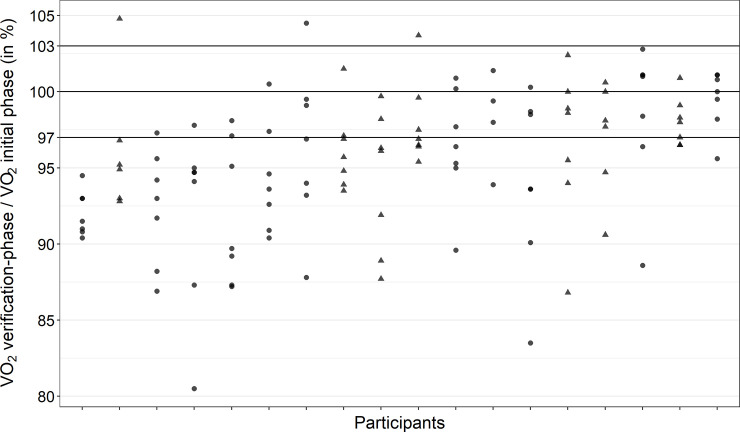
Ratio of $\dot{V}O_{2}\max$ achieved during the verification-phase divided by $\dot{V}O_{2}\max$ from the initial phase of the exercise test. Expressed as a percentage for all tests performed by the participants. Circles = males; triangles = females.

Four participants did not show a $\dot{V}O_{2}$-plateau in any of the seven tests (Fig 2). No athlete reached a $\dot{V}O_{2}$-plateau in all seven tests. In more than 60% of tests, the verification-$\dot{V}O_{2}$ was less than 97% of the $\dot{V}O_{2}\max$ from the initial phase of CPET (Fig 3). Average heart rate, blood lactate concentration and rating of perceived exertion at the end of the regeneration phase (i.e., immediately before the verification-phase) of all tests (n = 126) were 130 ± 13 bpm, 7.7 ± 2.8 mmol/L, and 9.5 ± 2.2. Linear regression analysis showed little evidence for training effects from the first to the last CPET ($\chi_{1}^{2}$ = 1.80, *p* = 0.179).

### Day-to-day variation

$\dot{V}O_{2}$-plateau appeared in 6 out of 34 tests, with an agreement regarding the appearance or non-appearance in 13 out of 17 participants. Gwet’s AC_1_ was 0.64 (95% CI: 0.22, 1.00). $\dot{V}O_{2}peak$ was confirmed in 11 out of 34 tests, with an agreement regarding verification or non-verification in 14 out of 17 participants. Gwet’s AC_1_ was 0.69 (95% CI: 0.31, 1.00). On the first day $\dot{V}O_{2}\max$ from the initial phase of the CPET was 4.00 ± 0.77 L/min and verification-$\dot{V}O_{2}$ was 3.78 ± 0.74 L/min on the second day the respective values were for $\dot{V}O_{2}\max$ 4.03 ± 0.81 L/min and for verification-$\dot{V}O_{2}$ 3.80 ± 0.84 L/min.

### Diurnal variation

In 56 out of the 108 tests performed at different times of day, $\dot{V}O_{2}peak$ was less than 200 mL/min lower than highest $\dot{V}O_{2}peak$ and were therefore used to analyse the effect of diurnal variation. A $\dot{V}O_{2}$-plateau appeared in 13 out of 56 tests with an agreement between the analysed tests in 10 out of 16 participants. Gwet’s AC_1_ was 0.71 (95% CI: 0.42, 0.99). $\dot{V}O_{2}\max$ was confirmed in 21 out of 56 tests with an agreement between the analysed tests in 4 out of 16 participants. Gwet’s AC_1_ was 0.07 (95% CI: -0.38, 0.52). $\dot{V}O_{2}\max$ from the initial phase of the CPET was 4.07 ± 0.78 L/min and verification-$\dot{V}O_{2}$ was 3.89 ± 0.77 L/min.

### Additional value of verification-phase

In 66% of the 56 tests analysed, performing a verification-phase added no value, in 32% it added uncertain value, and in 2% it added certain value in the determination of $\dot{V}O_{2}\max$.

### Influence of exercise duration at supra-peak load

The mean exercise duration at the supra-peak bout was 1.17 ± 0.34 minutes. There was a significant negative correlation between the duration at supra-peak load and the difference between $\dot{V}O_{2}\max$ from the initial phase of CPET and verification-$\dot{V}O_{2}$ (r = -0.363; p ≤ 0.001). In none of the tests that added uncertain value by performing a verification-phase, the supra-peak load was sustained for > 2 minutes.

## Discussion

The main results of this study are that $\dot{V}O_{2}$-plateau shows acceptable agreement for both day-to-day and diurnal variations. Analysing a $\dot{V}O_{2}$-plateau does not increase costs as compared to performing a verification-phase. However, a low incidence of individuals who have achieved a $\dot{V}O_{2}$-plateau was identified. The verification-phase protocol used in this study shows acceptable agreement only for day-to-day variations. Despite a slightly higher incidence of $\dot{V}O_{2}peak$ confirmation compared to the incidence of a $\dot{V}O_{2}$-plateau, the verification phase adds limited value in the determination of $\dot{V}O_{2}\max$ while simultaneously increasing the burden for participants and staff.

### Reliability of $\dot{V}O_{2}$-plateau and the verification-phase

The appearance or absence of a $\dot{V}O_{2}$-plateau seems to be robust to both day-to-day and diurnal variations in tests with comparable $\dot{V}O_{2}\max$. This robustness has meaningful implications for daily practice because tests can be performed over a relatively long time period around the time of peak performance without influencing the chance of $\dot{V}O_{2}$-plateau appearance. The $\dot{V}O_{2}$-plateau method carries no additional burden on the tested person, in contrast with the use of a verification-phase. However, because $\dot{V}O_{2}$-plateau does not appear in all participants, we recommend the use of secondary $\dot{V}O_{2}$-max criteria in those participants without $\dot{V}O_{2}$-plateau to ensure that $\dot{V}O_{2}peak$ is as close as possible to $\dot{V}O_{2}\max$ [5, 22]. In contrast to verification-phases, secondary $\dot{V}O_{2}\max$ criteria have been shown to be robust to both day-to-day and diurnal variations [22]. Furthermore, the simple use of RER ≥ 1.10 for example leads to a maximum underestimation of $\dot{V}O_{2}\max$ of 7% in 97.5% of 70 well-trained and 500 healthy participants [5, 9, 22]. This error is only slightly higher than the definitions used to verify $\dot{V}O_{2}\max$ by way of verification-phases (i.e. 3%–5.5%) [14, 25, 28].

### Influence of choice of the verification-phase protocol

For the purpose of this study we did not create a new verification protocol, but used an established one that has been repeatedly reported to be able to confirm incremental $\dot{V}O_{2}peak$ in this form or with small alterations [25, 29]. One could argue that choosing another protocol might have shown results that are more in favor of the verification-phase. However, in the following we illustrate common misconceptions and limitations regarding the $\dot{V}O_{2}$-verification concept to demonstrate that our choice of protocol is not the limiting factor. First, in contrast to many authors we chose a verification bout with supra-peak load, because using a submaximal load [30–32] do not correspond to the idea that a $\dot{V}O_{2}$-plateau can be provoked by a verification bout, as previously highlighted [3, 8]. Second, many previous authors [20, 30, 32–34] compared the verification-$\dot{V}O_{2}$ and ramp-$\dot{V}O_{2}$ on a group level and concluded that the verification was successful because there were no significant differences on a group level between the two $\dot{V}O_{2}$ values. Obviously not group averages need to be compared with each other, but the two $\dot{V}O_{2}$ values from each subject to define for each individual if $\dot{V}O_{2}\max$ was verified. Third, the duration of the supra-peak verification bout needs to be considered [16]. This is the most common limitation in analyzing and interpreting verification data. The majority of authors concluded that $\dot{V}O_{2}\max$ is reached if ramp-$\dot{V}O_{2}$ and verification-$\dot{V}O_{2}$ differ no more than a certain percentage despite a higher workload during the verification bout. However, this is only one possibility. The second explanation might be that the verification bout could not be sustained long enough to exceed the ramp-$\dot{V}O_{2}$, which becomes present in Fig 1. If the subject would have stopped after 1:15 min (as the average of subjects in this study did), the verification-$\dot{V}O_{2}$ would be 99% of the ramp-$\dot{V}O_{2}$ and it would be concluded that the verification bout was successful to verify $\dot{V}O_{2}\max$. However, the workload was sustained for 2:05 min which led to a verification-$\dot{V}O_{2}$ of 104% of the initial test indicating that $\dot{V}O_{2}\max$ was not reached in the previous test. Hill et al. [35] and Caputo and Denadai [36] showed that it takes a minimum of 2:00 min in trained athletes with fast $\dot{V}O_{2}$-kinetics and about 3:30 minutes in subjects with slow $\dot{V}O_{2}$-kinetics to reach their $\dot{V}O_{2}\max$. Therefore, we make a strong case that the duration of the supra-peak verification bout needs to be considered to ensure that $\dot{V}O_{2}\max$ can be achieved. The fact that the subjects in this study were not able to sustain the supra-peak load long enough to reach $\dot{V}O_{2}\max$ is clearly not limited to the protocol used in this study. In fact, irrespective of supra-peak intensity (ranging from 105% to 110%) or exercise mode (running or cycling) almost all studies showed shorter verification bouts as the required duration to reach $\dot{V}O_{2}\max$ [25, 28, 30, 34, 37, 38].

The stepwise increases to 50%, 70%, and 105% of PPO during the verification-phase has been promoted as an advantage in comparison with most protocols used previously [8, 13, 39] because it leads to higher $\dot{V}O_{2}$ uptake at the point at which the supra-peak load starts. Although we used this stepwise approach, many participants showed a lower verification-$\dot{V}O_{2}$ than that in the initial phase of CPET. In addition, we found a significant negative correlation (r = −0.363; p ≤ 0.001) between the duration at supra-peak load and the difference between $\dot{V}O_{2}\max$ from the initial phase of CPET and the verification-$\dot{V}O_{2}$. This negative correlation might be due to participants being incapable of maintaining the supra-peak load for a long enough time for $\dot{V}O_{2}$ to reach the initial phase value.

This is potentially caused by an insufficient duration of the regeneration phase. Remarkably, we chose a duration of 10 minutes, which is longer than that promoted in several other studies [14, 28, 30, 34, 37, 40]. Nevertheless, blood lactate concentrations at the end of the regeneration phase showed that the participants of our study had only moderately recovered. A longer regeneration duration might have led to better recovery [41], which could have increased the chance of verifying $\dot{V}O_{2}\max$ during the supra-peak verification phase. However, longer recovery phases would reduce practicability in clinical routine. Irrespective of this, in most other studies the verification bouts were longer sustained compared to our study despite they used a similar or even shorter recovery periods [29, 30, 37]. This indicates that an insufficient recovery is not the main cause for the preliminary termination of the verification bout.

Many participants did not reach a sufficient duration of the verification-phase, although we used a supra-peak load of 105% of PPO, which is relatively low in comparison with the 110% to 125% PPO values used in previous studies [14, 20, 25, 34, 42, 43]. However, most of the CPETs in these studies were performed with rather low incremental rates (< 20 W/min). As recently demonstrated by Iannetta et al. [16] ramp protocols with fast-increasing work rates lead to far higher peak work rates. This higher peak power output subsequently results in a higher work rate, which then needs to be sustained in a supra-peak verification-phase. Therefore, it is likely that the insufficient duration of the verification-phase was caused by the rather high incremental rate combined with a supra-peak verification load.

### Additional value of the verification-phase

A further neglected topic is the determination of additional value of performing a verification test. The incidence of a $\dot{V}O_{2}$-plateau usually ranges between 20%– 60% [4–6, 9, 32]. The plateau incidences of our present study were rather low but still in line with these values. If a $\dot{V}O_{2}$-plateau occurs, it is already clear that $\dot{V}O_{2}\max$ was reached and a verification bout does not provide any additional benefit. Likewise, if no $\dot{V}O_{2}$-plateau is reached but verification-$\dot{V}O_{2}$ is below 97% also no additional value is provided by the verification phase. In this study, the latter cases made up 66% of all tests. For 32% of tests it was uncertain if the verification test added value, because we do not know if $\dot{V}O_{2}\max$ was verified or if the verification bout was not sustained long enough to exceed the $\dot{V}O_{2}$ from the ramp test (see Fig 1). However, it should be kept in mind that performing a verification-phase increases time, costs, and effort required from investigators and participants in all tests. The benefit is therefore highly debatable, especially as almost none of the tests at least two minutes of supra-peak power were reached, which seems to be necessary to reliably verify $\dot{V}O_{2}\max$ with respect to the time constant of $\dot{V}O_{2}$-kinetics [16, 35, 36].

### Limitations

Limitations for our investigation include the method used to check for the occurrence of a $\dot{V}O_{2}$-plateau. Our used plateau definition is based on the assumption that $\dot{V}O_{2}$ increases 10 mL/min/W in the submaximal intensity domain [4]. As previously described, this is an oversimplification since the actual increase may differ slightly between participants [24]. Therefore, participants with a lower increase in the submaximal intensity domain may easier achieve the cut-off (125 mL/min) compared to participants with a steeper increase of $\dot{V}O_{2}$ [29]. However, the effect of this on the $\dot{V}O_{2}$ -plateau occurrence is rather small as recently described [4]. Therefore, it is unlikely that the use of a fixed cut-off considerable limits our findings.

The second limitation concerns the fact that nearly 50% of the incremental tests were excluded for the analysation of the effect of diurnal variations on the plateau occurrence and the verification procedure. As described in the method section this was necessary because it cannot be defined whether the diurnal variations in $\dot{V}O_{2}peak$ are caused by physiological reasons or by variations in pain tolerance and motivation.

## Conclusions

$\dot{V}O_{2}$-plateau showed an acceptable level of agreement for day-to-day and diurnal variations without additional burden for participants. However, the $\dot{V}O_{2}$-plateau incidence was rather low, which is in line with previous studies [5, 6, 32]. In contrast, the verification-phase method shows acceptable agreement only for the day-to-day variation. Furthermore, this method provided certain additional value in 2% of tests only and, therefore, it hardly justifies the increased participant burden, time, and financial costs required. This low rate of additional value is likely caused by fact that a verification bout, which is performed at work rate above PPO of a previous incremental test with a high incremental rate cannot be performed for sufficient to duration to allow $\dot{V}O_{2}$ to rise to the maximum. We conclude that the verification protocol used in this study with athletes can be omitted.

## Supporting information

S1 Data (7Z)
